# Retrospective single center cohort study: effect of intensive home hemodialysis on right ventricular systolic pressure and clinical outcomes

**DOI:** 10.1186/s12882-020-02159-z

**Published:** 2020-11-25

**Authors:** Michael Girsberger, Christopher T. Chan

**Affiliations:** Division of Nephrology, University Health Network, 200 Elizabeth Street, 8N room 846, Toronto, ON M5G 2C4 Canada

**Keywords:** Intensive home hemodialysis, Right ventricular systolic pressure, Pulmonary hypertension, Clinical outcomes

## Abstract

**Background:**

Increased right ventricular systolic pressure (RVSP), a surrogate marker for pulmonary hypertension, is common in patients with end-stage kidney disease. Limited data suggest improvement of RVSP with intensive dialysis, but it is unknown whether these improvements translate to better clinical outcomes.

**Methods:**

We conducted a retrospective single center cohort study at the Toronto General Hospital. All patients who performed intensive home hemodialysis (IHHD) for at least a year between 1999 and 2017, and who had a baseline as well as a follow-up echocardiogram more than a year after IHHD, were included. Patients were categorized into two groups based on the RVSP at follow-up: elevated (≥ 35 mmHg) and normal RVSP. Multivariate and cox regression analyses were done to identify risk factors for elevated RVSP at follow-up and reaching the composite endpoint (death, cardiovascular hospitalization, treatment failure), respectively.

**Results:**

One hundred eight patients were included in the study. At baseline, 63% (68/108) of patients had normal RVSP and 37% (40/108) having elevated RVSP. After a follow-up of 4 years, 70% (76/108) patient had normal RVSP while 30% (32/108) had elevated RVSP. 8 (10%) out of the 76 patients with normal RVSP and 15 (47%) out of the 32 patients with elevated RVSP reached the composite endpoint of death, cardiovascular hospitalization or technique failure. In a multivariate analysis, age, diabetes and smoking were not associated with elevated RVSP at follow-up. Elevated RVSP at baseline was not associated with a higher likelihood in reaching the composite endpoint or mortality.

**Conclusion:**

Mean RVSP did not increase in patients on IHHD over time, and maintenance of normal RVSP was associated with better clinical outcomes.

**Supplementary Information:**

The online version contains supplementary material available at 10.1186/s12882-020-02159-z.

## Background

Right ventricular dysfunction and pulmonary hypertension (PH) are common in patients on conventional in-center hemodialysis) and have been reported in up to 50% [1]. A study recently published by Santosh et al. showed an increase over time in pulmonary artery systolic pressure in in-center hemodialysis as well as in peritoneal dialysis (PD) [2]. Both right ventricular dysfunction and PH, have been shown to be associated with adverse outcomes [3–6]. Therefore, possible interventions to prevent PH or improve right ventricular parameters in patients with ESRD are of great interest. Studies of right ventricular function and pulmonary hypertension in patients undergoing intensive dialysis are limited. One study reported reduced left and right ventricular end systolic volumes in patients undergoing frequent in-center hemodialysis for 12 months [7]. In another study, 37 patients changing from in-center hemodialysis (12 h/week) to nocturnal in center dialysis (21 h/week) had a significant reduction of right ventricular end-systolic volume index after 1 year [8]. There is a paucity of longitudinal studies detailing the impact of intensive hemodialysis on right ventricular function and structure.

### Objective

The aim of this study was to investigate the longitudinal effect of intensive hemodialysis on right ventricular systolic pressure (RVSP) as a surrogate marker for PH. Additionally, we investigated possible risk factors for elevated RVSP over time and associated clinical outcomes. We hypothesized that RVSP in patients undergoing intensive HD would not increase at follow-up due to effective extracellular volume control. We ascertained if changes in RVSP correlate with changes in left atrial pressures. Further, we aimed to examine if other factors contributed to the development of elevated RVSP.

## Methods

We performed a retrospective single center cohort study at Toronto General Hospital (TGH). All patients who initiated IHHD between 1 January 1999 and 31 January 2017, and had an echocardiogram at dialysis initiation and a follow-up echocardiogram at least a year after IHHD initiation. Patients on IHHD for less than a year or with missing echocardiographic information at baseline or follow-up were excluded. Patients with tricuspid regurgitation were excluded due to missing RVSP measurement. The study was conducted with adherence to the Declaration of Helsinki and approved by the University Health Network Research Ethics Board.

IHHD was defined as ≥16 h of dialysis per week based on the prescribed regimen at the end of home hemodialysis training. A standard prescription consisted of five nights or more for at least 7 hours (35 h per week).

Echocardiograms were performed before a hemodialysis session at baseline when initiating IHHD and yearly thereafter. RVSP was measured by estimating the right ventricle–right atrial pressure gradient from the tricuspid regurgitation (TR) maximal velocity. Normal RVSP was defined as < 35 mmHg [9, 10]. Normal left arterial pressure was defined as ≤15 mmHg and normal left ventricular ejection fraction (LVEF) was defined as ≥55% [11] applying the modified biplane Simpson’s rule. Baseline echocardiogram and the last available follow-up echocardiogram while on IHHD were used for analysis.

Patients were categorized into two groups based on RVSP at baseline: normal RVSP (< 35 mmHg) and abnormal RVSP (≥35 mmHg).

The primary outcome was the proportion of patients with normal and abnormal RVSP at follow-up, respectively. Secondary outcomes included the composite endpoint of technique failure, cardiovascular events or death and possible risk factors for this composite endpoint. Technique failure was defined as permanently switching to another renal replacement therapy modality such as peritoneal dialysis or in-center hemodialysis. Cardiovascular events consisted of stroke, myocardial infarction, unstable angina, arrhythmia and heart failure.

Baseline patient characteristics were collected at the start of IHHD. The following variables were collected: demographic data (age, race, gender), body mass index (BMI), cause of end-stage kidney disease (ESKD), type of renal replacement therapy prior to intensive hemodialysis (not on dialysis, non-intensive hemodialysis, peritoneal dialysis (PD), renal transplantation), initial vascular access type and access type at follow-up (central venous catheter (CVC), arteriovenous fistula (AVF), arteriovenous graft (AVG)), fistula or graft flow if present, duration of ESKD, RVSP on baseline echocardiogram, co-existing medical conditions (diabetes, smoking history, hypertension, coronary artery disease (CAD), peripheral vascular disease) and blood pressure medications at baseline and after 1 year.

Demographic data, time of dialysis initiation and cause of end-stage kidney disease were abstracted form TGH IHHD database. Information on comorbidities, type of renal replacement therapy prior to intensive hemodialysis and initial access type were manually collected from patient’s medical records. Duration of ESKD was defined from initiation of any renal replacement therapy (RRT) until inclusion in into the study. If the exact date of RRT initiation was not available the midpoint of the indicated month or year was used, respectively. CAD was defined as previous myocardial infarction, coronary artery bypass graft or established CAD on imaging. Peripheral vascular disease was defined as vascular calcification on imaging with symptoms of peripheral hypoperfusion.

### Statistical analysis

Baseline characteristics and covariates were described using mean and standard deviation or proportions were used. Continuous variables were compared using the Student t or Wilcoxon rank sum test. Chi-square and McNemar test were used to compare proportions. Linear regression analysis was used to investigate correlation of RVSP and left atrial pressure. Multivariate logistic regression analysis was conducted to investigate risk factors for increased RVSP at follow-up. To investigate the impact of different clinical variables on the composite endpoint, a Cox regression model was used. A Kaplan-Meier plot was used to evaluate the time to composite endpoint in patients with normal RVSP and elevated RVSP at follow-up. All analyses were performed using STATA version SE 14.2 (StataCorp, USA).

## Results

**Fig. 1:**
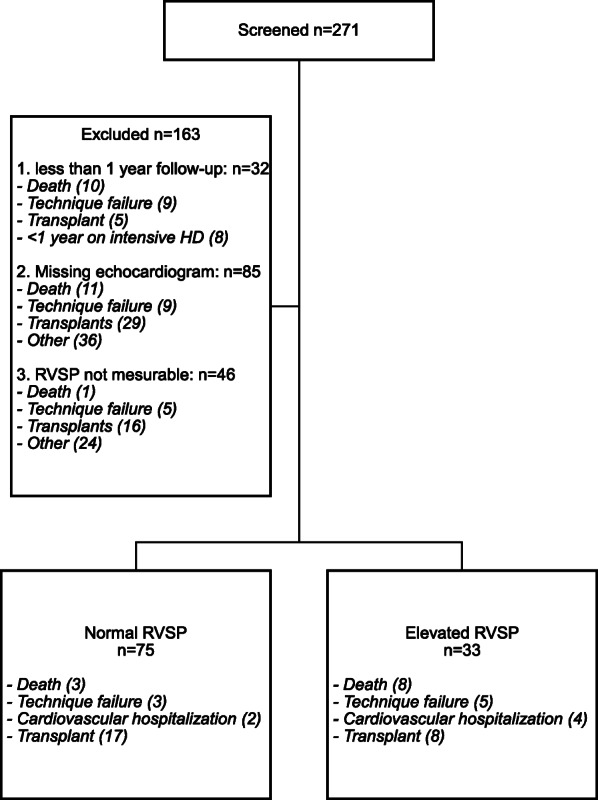
Flow diagram (HD: hemodialysis, RVSP: right ventricular systolic pressure)

The study cohort is defined in Fig. 1. Of 271 patients, 108 were included for final analysis. Baseline characteristics are summarized in Table 1.

**Table 1:** Baseline Characteristics (SD: standard deviation; ESKD: end-stage renal disease; CVC: central venous catheter; AVF: arteriovenous fistula; AVG: arteriovenous graft; RAAS: renin angiotensin aldosterone system; CCB: calcium channel blocker)

	All patients (*n* = 108)
Age, years, mean ± SD	45 ± 13
Male sex, n (%)	67 (62)
Race, n (%)	
- White	58 (54)
- Asian	15 (14)
- Black	14 (13)
- Other	20 (19)
BMI, kg/m2, mean ± SD	24.5 ± 5.5
Cause of ESRD, n (%)	
- Diabetic nephropathy	12 (11)
- Glomerulonephritis	44 (40)
- Hypertensive nephrosclerosis	7 (6)
- Polycystic kidney disease	10 (9)
- Other	35 (32)
Type of renal replacement therapy prior to intensive hemodialysis, n (%)	
- Chronic kidney disease not on dialysis	32 (30)
- Non-intensive hemodialysis	39 (36)
- Peritoneal dialysis	11 (10)
- Renal transplantation	26 (24)
Initial access type, n (%)	
- CVC	55 (51)
- AVF	43 (40)
- AVG	10 (9)
Duration of ESKD, years, median (IQR)	2 (0.3–12)
Hours of dialysis per week, mean ± SD	35 ± 7.3
Total follow up in years, mean ± SD	4.0 ± 2.2
Time between echocardiograms in years, mean ± SD	3.3 ± 2.0
Co-existing medical conditions, n (%)	
- Diabetes	27 (25)
- Smoking history	20 (19)
- Hypertension	93 (86)
- Coronary artery disease	10 (9)
- Peripheral vascular disease	6 (6)
- Stroke	6 (6)
Baseline blood pressure therapy	
- Number of antihypertensives, mean ± SD	1.8 ± 1.3
- Patients on > 2 antihypertensives, n (%)	28 (26)
- RAAS blockade/CCB/β-blocker, %	47/52/51
1-Year blood pressure therapy	
- Number of antihypertensives, mean ± SD	0.8 ± 1.0
- Patients on > 2 antihypertensives, n (%)	6 (6)
- RAAS blockade/CCB/β-blocker, %	17/17/43

### Normal vs elevated RVSP at follow-up

At baseline, 63% (68/108) of patients had normal RVSP and 37% (40/108) having elevated RVSP.

**Fig. 2:**
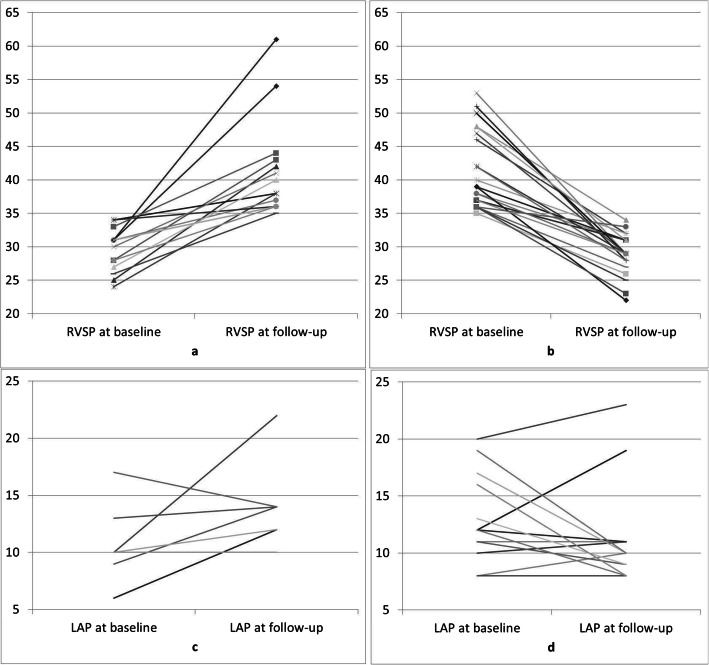
Patients with changing RVSP from elevated to normal (**a**) and normal to elevated (**b**) and correlating change in LAP (**c** and **d**)

Of the 68 patients with normal RVSP at baseline, 22% (15/68) had elevated RVSP at follow-up (Fig. 2a). Conversely, of the 40 patients with elevated RVSP at baseline, 58% (23/40) had normal RVSP at follow-up (Fig. 2b). Overall, mean RVSP did not significantly differ between baseline and follow-up.

At a mean follow-up of 4 years, 70% (76/108) patient had normal RVSP while 30% (32/108) had elevated RVSP (Table 2). Characteristics of patients with elevated and normal RVSP at follow-up, respectively, are shown in Table 2. Coronary artery disease (3% vs 12%; *p* = 0.04) and peripheral vascular disease (3% vs 12%; *p* = 0.04) were both significantly more prevalent in patients with elevated RVSP at follow-up. All other baseline characteristics including blood pressure therapy at baseline and after 1 year on IHHD did not differ between patients with normal and elevated RVSP at follow-up.

**Table 2:** Characteristics of patients with elevated RVSP and normal RVSP at follow-up (SD: standard deviation; ESKD: end-stage kidney disease; CVC: central venous catheter; AVF: arteriovenous fistula; AVG: arteriovenous graft; RAAS: renin angiotensin aldosterone system; CCB: calcium channel blocker)

	Normal RVSP (*n* = 76)	Elevated RVSP (*n* = 32)	*p*-value
Age, years, mean ± SD	44 ± 12.5	49 ± 14.8	NS
Male sex, n (%)	48 (64)	19 (58)	NS
Race, n (%)			
- White	40 (53)	18 (55)	NS
- Asian	9 (12)	6 (18)	NS
- Black	12 (16)	2 (6)	NS
- Other	14 (19)	7 (21)	NS
BMI, kg/m2, mean ± SD	24.8 ± 5.7	23.7 ± 4.9	NS
Cause of ESRD, n (%)			
- Diabetic nephropathy	8 (11)	4 (12)	NS
- Glomerulonephritis	29 (39)	15 (46)	NS
- Hypertensive nephrosclerosis	4 (5)	3 (9)	NS
- Polycystic kidney disease	7 (9)	3 (9)	NS
- Other	27 (36)	8 (24)	NS
Type of renal replacement therapy prior to intensive hemodialysis, n (%)			
- Chronic kidney disease not on dialysis	23 (31)	9 (27)	NS
- Non-intensive hemodialysis	27 (36)	12 (36)	NS
- Peritoneal dialysis	5 (7)	6 (18)	NS
- Renal transplantation	20 (27)	6 (18)	NS
Initial access type, n (%)			
- CVC	40 (53)	15 (46)	NS
- AVF	26 (35)	17 (52)	NS
- AVG	9 (12)	1 (3)	NS
Duration of ESKD, years, median (IQR)	1.7 (0.3–12)	2.6 (0.2–14)	NS
Hours of dialysis per week, mean ± SD	36 ± 8.0	34 ± 7.8	NS
Total follow up in years, mean ± SD	3.9 ± 2.2	4.1 ± 2.3	NS
Time between echocardiograms in years, mean ± SD	3.2 ± 2.8	3.4 ± 2.2	NS
Co-existing medical conditions, n (%)			
- Diabetes	17 (23)	10 (30)	NS
- Smoking history	12 (16)	8 (24)	NS
- Hypertension	65 (87)	28 (85)	NS
- Coronary artery disease	5 (7)	5 (15)	NS
- Peripheral vascular disease	2 (3)	4 (12)	0.04
- Stroke	2 (3)	4 (12)	0.04
Baseline blood pressure therapy			
- Number of antihypertensives, mean ± SD	1.9 ± 1.3	1.5 ± 1.2	NS
1-Year blood pressure therapy			
- Number of antihypertensives, mean ± SD	0.8 ± 1.0	0.8 ± 0.9	NS

### RVSP status at follow-up and clinical outcomes

Eight (10%) out of the 76 patients with normal and 15 (47%) out of the 32 with elevated RVSP at follow-up reached the composite endpoint of death, cardiovascular hospitalization or technique failure. Three (4%) and eight (25%) deaths occurred in patients with normal and elevated RVSP at follow-up, respectively. Three (4%) patients with normal and five (16%) patients with elevated RVSP developed technique failure. Hospitalization due to cardiovascular events was observed in two patients (3%) with normal (stroke and arrhythmia) and four (13%) with elevated (arrhythmia, unstable angina, stroke, myocardial infarction) RVSP at follow-up. Out of the entire cohort, 25 (23%) patients received a kidney transplant during the study period, 17 (23%) of patients with normal and 8 (24%) with elevated RVSP at follow-up.

**Fig. 3:**
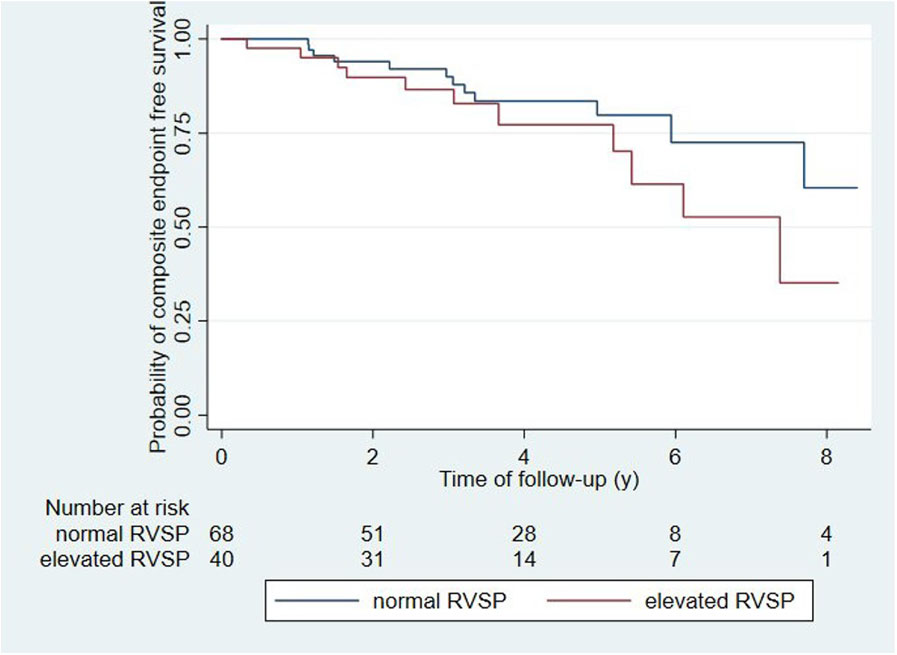
Composite end point-free survival of patients with elevated and normal RVSP at baseline, respectively. Elevated RVSP at baseline was not associated with a higher risk to reach the composite endpoint (log rank: p = 0.18)

**Fig. 4:**
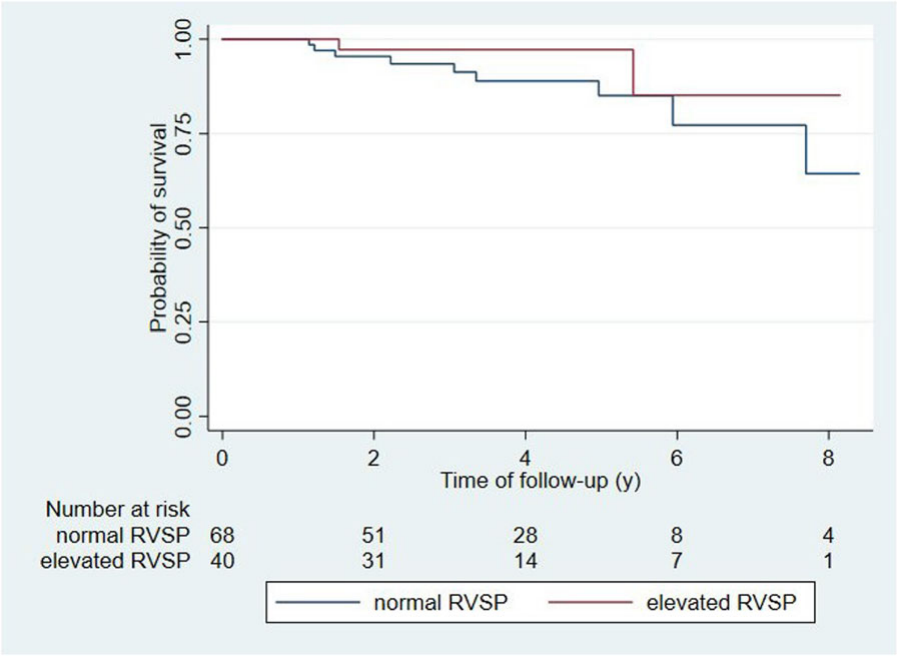
Patient survival in patients with elevated and normal RVSP at basline, respectively. Survival did not differ between those two groups (log-rank: *p* = 0.28)

In univariate logistic regression analyses, only elevated RVSP at baseline was significantly associated with elevated RVSP at follow-up (OR 2.4 [95% CI 1.0–5.5]). In multivariate analysis, none of the investigated parameters were significantly associated with elevated RVSP at follow-up (Table 3). Kaplan-Meier curves with log rank test showed no difference for event free survival in patients with normal RVSP at baseline in regard to both the composite endpoint (*p* = 0.18; Fig. 3) and death (*p* = 0.01; Fig. 4). In an unadjusted Cox proportional hazards analysis, patients with elevated RVSP at baseline were also not at higher risk of reaching the composite endpoint (HR 1.7 [95% CI 0.8–3.9]). Patients with elevated RVSP at follow-up remained at a significantly higher risk of reaching the composite endpoint when adjusted for age and diabetes (HR 4.4 [95% CI 1.8–10.6]). Diabetes at baseline was associated with a higher risk in unadjusted analysis (HR 2.5 [95% CI 1.1–5.9]), but not after adjusting for age and elevated RVSP at baseline (HR 2.2 [95% CI 0.9–5.4]) (Table 4). Patient survival was significantly shorter in patients with elevated RVSP in unadjusted (HR 6.4 [95% CI 1.7–24.1]) as well as adjusted (HR 6.2 [95% CI 1.6–23.9]) analyses (Table 5).

**Table 3:** Multivariate analysis for elevated RVSP at follow-up (LVEF: left ventricular ejection fraction; LAP: left atrial pressure; RVSP: right ventricular systolic pressure)

Variable	Unadjusted OR (95% CI)	Adjusted OR (95% CI)
Age	1.03 (0.99–1.06)	1.02 (0.98–1.05)
Reduced LVEF	2.16 (0.66–7.01)	1.75 (0.50–6.16)
Elevated LAP	2.00 (0.51–7.89)	1.68 (0.38–7.45)
Coronary heart disease	2.50 (0.67–9.31)	1.42 (0.32–6.27)
Elevated RVSP at baseline	2.40 (1.04–5.57)	2.04 (0.85–4.91)
Smoking history	1.65 (0.60–4.53)	1.52 (0.52–4.43)
Having fistula or graft	1.42 (0.61–3.31)	1.50 (0.61–3.64)
Fistula/Graft flow	1.00 (0.99–1.00)	1.00 (0.99–1.00)

**Table 4:** Cox proportional hazards analysis of the risk of the composite end point of death, technique failure and CV-related hospitalization

Variable	Unadjusted HR (95% CI)	Adjusted HR (95% CI)
Normal vs elevated RVSP at baseline	1.7 (0.8–3.9)	1.4 (0.6–3.4)
Diabetes	2.6 (1.1–5.9)	2.2 (0.9–5.4)
Age	1.0 (0.9–1.1)	1.0 (0.9–1.1)

**Table 5:** Cox proportional hazards analysis for the risk of death

Variable	Unadjusted HR (95% CI)	Adjusted HR (95% CI)
Normal vs elevated RVSP at baseline	0.4 (0.1–2.1)	0.3 (0.1–1.5)
Diabetes	1.6 (0.4–5.9)	1.5 (0.4–6.0)
Age	1.0 (0.9–1.1)	1.0 (0.9–1.1)

We also conducted a sensitivity analysis using a cut-off of ≥40 mmHg for elevated RVSP with similar results (Supplementary Material Table S1, 2, 3, Fig. S1–2).

## Discussion

While there are several studies on right ventricular geometry and function [7, 12], our study is the first to report on RVSP over time in patients undergoing IHHD. In-center hemodialysis is a well described risk factor for new onset or worsening PH [13–15]. Our study shows that RVSP did not increase over time in a large cohort of patients undergoing IHHD. Furthermore, we also present the first study showing the clinical implications of normal RVSP in patients on IHHD. Composite end point free survival as well as overall survival did not differ between patients with elevated RVSP at baseline and normal RVSP.

Overall, the proportion of patient with elevated RVSP did not increase over time (37% vs 30%) in our cohort undergoing IHHD, while new onset or worsening RVSP is a known risk in patients undergoing i in-center hemodialysis [13–15]. In fact, elevated RVSP normalized in the majority of patients on IHHD over time (58%). In contrast, new onset PH was only observed in a minority (22%) (Fig. 2 a,b). This has several clinical implications.

**Fig. 5:**
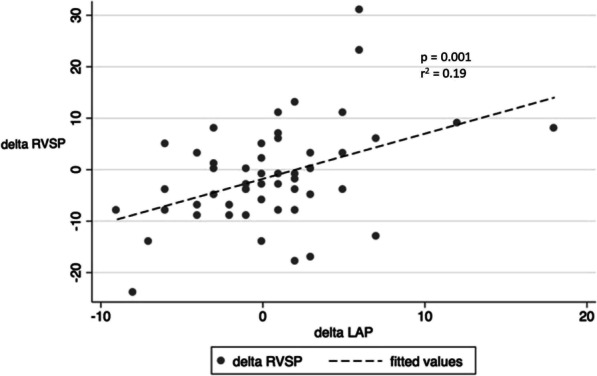
Correlation of change in RVSP and LAP over time in all patients

Right heart cardiac output (volume) and pulmonary vascular resistance determine pulmonary arterial pressure. Dialysis specific risk factors for pulmonary hypertension are mostly volume related and include arteriovenous (AV) access with increased venous volume return, left ventricular dysfunction and hypervolemia. While we had data on AV access status and left ventricular ejection fraction, we did not have information on volume status nor diastolic heart failure. Increased left atrial pressure is indicative for post-capillary PH due to left heart dysfunction, systolic and diastolic. Left atrial pressure was not associated with elevated RVSP in our study (Table 3) nor did left atrial pressure differ between patients with normal and elevated RVSP at follow-up (11 vs 13 mmHg, *p* = 0.08). However, there was a correlation of change in RVSP and left atrial pressure over time (Fig. 5). Similar trends were noted in the subgroups as noted in Fig. 2. Taken together, it is reasonable to speculate that a possible association of amelioration of RVSP is with better volume control.

AV access, AV flow and LVEF showed no association with elevated RVSP at follow-up (Table 3). However, with regard to LVEF, only three patients of the entire cohort (3%) had severely reduced LVEF (< 35%) at follow-up, making it difficult to draw conclusions regarding the impact of advanced heart failure on RVSP in this study. As better volume control along with lower blood pressure [16] are well-known advantages of IHHD, it is presumably accountable for some favorable effects with regard to RVSP in our study.

Challenged with increasing volume, the pulmonary vascular bed is usually able to adapt by decreasing vascular resistance with only little or no increase in pulmonary arterial pressure. Thereby, increased resistance of the pulmonary vascular bed is key in development of PH [13]. Several mechanisms are thought to affect pulmonary arterial vascular resistance in patients with CKD and ESRD giving possible explanations for our findings. Changes in vascular endothelium metabolism [17, 18] and vascular calcification [19, 20] leading to increased vascular resistance have been reported in patients with ESKD undergoing dialysis and may be a reason for increased pulmonary vascular resistance. IHHD has been shown to improve peripheral vascular smooth muscle cell biology [21] and lower peripheral resistance. The same mechanism could positively affect the pulmonary vasculature. Better control of calcium phosphate hemostasis in IHHD patients [22] with could also have additional beneficial effects on vascular resistance in the pulmonary vasculature. Furthermore, left heart failure can increase pulmonary pressure not only by post-capillary PH; chronically elevated left ventricular filling pressures can also reduce pulmonary vascular resistance [23]. IHHD has been shown to improve left ventricular hypertrophy (LVH) as well as preserve LVEF over several years [24, 25]. Possible mechanisms for these findings have been discussed in detail previously [24], but are generally due to better volume control, reduced cardiac stunning, better metabolic control and decrease in peripheral vascular resistance. Finally, obstructive sleep apnea with nocturnal hypoxemia has been shown to be an important risk factor for PH and is highly prevalent in patients with ESRD [26, 27]. IHHD could therefore by improving sleep apnea [28] have a positive effect on PH.

In conclusion, there are several possible mechanisms of how IHHD may be protective for pulmonary arterial pressure by optimizing volume status, improving left heart geometry and function, decreasing pulmonary vascular resistance and improving sleep apnea.

### Strength and limitations

Our study has several strengths. We are the first to report on serial RVSP measurements over a mean follow-up of over 4 years. While single center design can decrease generalizability, patient management is less heterogeneous. Furthermore, by having baseline echocardiograms we were able to analyze RVSP over time. Finally, we could correlate RVSP with a composite endpoint as well as death due to detailed documentation of clinical endpoints such as hospitalization and technique failure. While we acknowledge that there are non-clinical reasons for technique failure such as patient’s choice, it is indicative for frailty in a self-care setting.

There are several limitations to our study. Patients suitable for IHHD tend to differ from patients undergoing in-center hemodialysis. A study comparing baseline characteristics from the Frequent Hemodialysis Network (FHN) daily and nocturnal cohorts to the USRDS cohort showed that patient undergoing frequent dialysis tend to be younger, are predominantly male, and differ in the prevalence of underlying kidney disease (more glomerulonephritis and less diabetic nephropathy) [29]. Prevalence of CVC was reported to be higher in patients undergoing nocturnal dialysis [29]. While some of these characteristics such as diabetes and AV access have been described as risk factors for elevated RVSP, the pathophysiology for PH in ESRD seems to be complex and additional risk factors such as endothelial dysfunction, and metabolic and hormonal changes play an important role in its development.

Using only one echocardiogram at follow-up as well as inter-observer variability are possible sources of undetected fluctuations of RVSP. While we tried to correct for multiple co-morbidities and clinical conditions, we cannot exclude residual confounding. Small sample size, limited generalizability and missing control group are additional limitations due to the retrospective single center design. While we used treatment failure as part of the composite endpoint as a marker for frailty, we acknowledge that there are non-clinical reasons for changing treatment such as patient choice. Finally, although measuring RVSP with echocardiograms is non-invasive and economical, the gold standard for measuring PH is by right heart catheterization.

## Conclusion

In our retrospective study, the number of patients with elevated RVSP did not increase over a mean follow-up of 4 years. Furthermore, the majority of patients with elevated RVSP at baseline had normalization of RVSP at follow-up and IHHD might therefore be an option for patients with ESKD and elevated pulmonary arterial pressure.

## Supplementary Information

**Additional file 1: Table S1.** Characteristics of patients with elevated RVSP defined as a cut-off of ≥40 mmHg and normal RVSP at follow-up.

**Additional file 2: Table S2.** Multivariate analysis for elevated RVSP at follow-up defined as ≥40 mmHg.

**Additional file 3: Table S3.** Cox proportional hazards analysis of the risk of the composite end point of death, technique failure and CV-related hospitalization using cut off of ≥40% for elevated RVSP.

**Additional file 4: Figure S1.** Flow diagram (≥40 mmHg).

**Additional file 5: Figure S2.** Composite endpoint free survival in patients with elevated (≥40 mmHg) and normal RVSP at base line, respectively.

## Data Availability

The datasets used and/or analysed during the current study are available from the corresponding author on reasonable request.

## Abbreviations

PH: Pulmonary hypertension
RVSP: Right ventricular systolic pressure
EF: Ejection fraction
IHHD: Intensive home hemodialysis
CHF: Congestive heart failure
PVR: Peripheral vascular resistance
AVF: Arterio-venous fistula
LVH: Left ventricular hypertrophy
CHD: Coronary heart disease
ESRD: End-stage renal disease
PD: Peritoneal dialysis
HD: Hemodialysis
TR: Tricuspid regurgitation
BMI: Body mass index
CVC: Central venous catheter
AVG: Arteriovenous graft
CAD: Coronary artery disease
RRT: Renal replacement therapy
HR: Hazard ration
CI: Confidence interval
AV: Arteriovenous
