# Supplementary material for: Retrospective single center cohort study: effect of intensive home hemodialysis on right ventricular systolic pressure and clinical outcomes
Source: BMC Nephrol. 2020 Nov 25;21:508. doi: 10.1186/s12882-020-02159-z (PMC7687753; doi:10.1186/s12882-020-02159-z)
Supplement: Supplementary file 1 — Additional file 1: Table S1. Characteristics of patients with elevated RVSP defined as a cut-off of ≥40 mmHg and normal RVSP at follow-up. [file 12882_2020_2159_MOESM1_ESM.docx]

|  | Normal RVSP  (n=93) | Elevated RVSP  (n=15) | p-value |
| --- | --- | --- | --- |
| Age, years, mean ± SD | 45 ± 13.1 | 47 ± 14.8 | 0.57 |
| Male sex, n (%) | 59 (63) | 8 (53) | 0.45 |
| Race, n (%) |  |  |  |
| - White | 50 (54) | 8 (53) | 0.98 |
| - Asian | 13 (14) | 2 (13) | 0.95 |
| - Black | 13 (14) | 1 (7) | 0.43 |
| - Other | 17 (18) | 4 (27) | 0.45 |
| BMI, kg/m2, mean ± SD | 24.4 ± 5.6 | 24.9 ± 4.8 | 0.76 |
| Cause of ESRD, n (%) |  |  |  |
| - Diabetic nephropathy | 10 (11) | 2 (13) | 0.77 |
| - Glomerulonephritis | 35 (38) | 9 (60) | 0.10 |
| - Hypertensive nephrosclerosis | 5 (5) | 2 (13) | 0.25 |
| - Polycystic kidney disease | 9 (10) | 1 (7) | 0.71 |
| - Other | 34 (37) | 1 (7) | 0.02 |
| Type of renal replacement therapy prior to intensive hemodialysis, n (%) |  |  |  |
| - Chronic kidney disease not on dialysis | 26 (28) | 6 (40) | 0.34 |
| - Non-intensive hemodialysis | 35 (38) | 4 (27) | 0.41 |
| - Peritoneal dialysis | 10 (11) | 1 (7) | 0.62 |
| - Renal transplantation | 22 (24) | 4 (27) | 0.80 |
| Initial access type, n (%) |  |  |  |
| - CVC | 47 (51) | 8 (53) | 0.84 |
| - AVF | 37 (40) | 6 (40) | 0.99 |
| - AVG | 9 (10) | 1 (7) | 0.71 |
| Duration of ESRD, years, mean ± SD | 7.3 ± 9.06 | 4.8 ± 6.7 | 0.30 |
| Hours of dialysis per week, mean ± SD | 35 ± 7.5 | 36 ± 6.0 | 0.64 |
| Total follow up in years, mean ± SD | 4.0 ± 3.6 | 3.7 ± 2.5 | 0.63 |
| Time between echocardiograms in years, mean ± SD | 3.3 ± 2.0 | 3.1 ± 2.0 | 0.76 |
| Co-existing medical conditions, n (%) |  |  |  |
| - Diabetes | 23 (25) | 4 (27) | 0.87 |
| - Smoking history | 17 (18) | 3 (20) | 0.89 |
| - Hypertension | 79 (85) | 14 (93) | 0.38 |
| - Coronary artery disease | 8 (9) | 2 (13) | 0.56 |
| - Peripheral vascular disease | 3 (3) | 3 (20) | <0.01 |
| - Stroke | 5 (5) | 1 (7) | 0.84 |
| Baseline blood pressure therapy |  |  |  |
| - Number of antihypertensives, mean ± SD | 1.8 ± 1.3 | 1.5 ± 0.9 | 0.42 |
| - Patients on >2 antihypertensives, n (%) |  |  |  |
| - RAAS blockade/CCB/β-blocker, % |  |  |  |
| 1-Year blood pressure therapy |  |  |  |
| - Number of antihypertensives, mean ± SD | 0.8 ± 1.0 | 0.9 ± 0.8 | 0.79 |
| - Patients on >2 antihypertensives, n (%) |  |  |  |
| - RAAS blockade/CCB/β-blocker, % |  |  |  |

Table S1: Characteristics of patients with elevated RVSP defined as a cut-off of ≥ 40mmHg and normal RVSP at follow-up
