# Supplementary material for: Retrospective single center cohort study: effect of intensive home hemodialysis on right ventricular systolic pressure and clinical outcomes
Source: BMC Nephrol. 2020 Nov 25;21:508. doi: 10.1186/s12882-020-02159-z (PMC7687753; doi:10.1186/s12882-020-02159-z)
Supplement: Supplementary file 2 — Additional file 2: Table S2. Multivariate analysis for elevated RVSP at follow-up defined as ≥40 mmHg. [file 12882_2020_2159_MOESM2_ESM.docx]

| Variable | Unadjusted OR (95% CI) | p value | Adjusted OR (95% CI) | p value |
| --- | --- | --- | --- | --- |
| Age | 1.01(0.97-1.06) | 0.57 | 1.01 (0.97-1.05) | 0.69 |
| Reduced LVEF | 2.08 (0.49-8.63) | 0.31 | 2.50 (0.54-11.51) | 0.24 |
| Elevated LAP | 2.00 (0.36-11.11) | 0.36 | 2.72 (0.43-17.17) | 0.29 |
| Coronary heart disease | 1.63 (0.31-8.56) | 0.56 | 1.25 (0.19-8.13) | 0.81 |
| Elevated RVSP at baseline | 3.08 (0.91-10.45) | 0.07 | 2.77 (0.59-12.96) | 0.20 |
| Smoking history | 1.10 (0.28-4.34) | 0.89 | 0.83 (0.16-4.30) | 0.83 |
| Having fistula or graft | 2.33 (0.59-9.10) | 0.22 | 1.99 (0.49-8.05) | 0.33 |
| Fistula flow | 1.00 (0.99-1.00) | 0.46 | 1.00 (0.99-1.00) | 0.07 |

Table S2: Multivariate analysis for elevated RVSP at follow-up defined as ≥40 mmHg
