# Supplementary material for: Retrospective single center cohort study: effect of intensive home hemodialysis on right ventricular systolic pressure and clinical outcomes
Source: BMC Nephrol. 2020 Nov 25;21:508. doi: 10.1186/s12882-020-02159-z (PMC7687753; doi:10.1186/s12882-020-02159-z)
Supplement: Supplementary file 3 — Additional file 3: Table S3. Cox proportional hazards analysis of the risk of the composite end point of death, technique failure and CV-related hospitalization using cut off of ≥40% for elevated RVSP. [file 12882_2020_2159_MOESM3_ESM.docx]

| Variable | Unadjusted HR (95% CI) | Adjusted HR (95% CI) |
| --- | --- | --- |
| elevated vs normal RVSP | 1.8 (0.7-4.9) | 1.5 (0.5-4.2) |
| Diabetes | 2.6 (1.1-5.9) | 2.2 (0.9-5.5) |
| Age | 1.0 (0.9-1.0) | 1.01 (0.9-1.0) |

Table S3: Cox proportional hazards analysis of the risk of the composite end point of death, technique failure and CV-related hospitalization using cut off of ≥40% for elevated RVSP at baseline
