# Supplementary figures and images for: Retrospective single center cohort study: effect of intensive home hemodialysis on right ventricular systolic pressure and clinical outcomes
Source: BMC Nephrol. 2020 Nov 25;21:508. doi: 10.1186/s12882-020-02159-z (PMC7687753; doi:10.1186/s12882-020-02159-z)

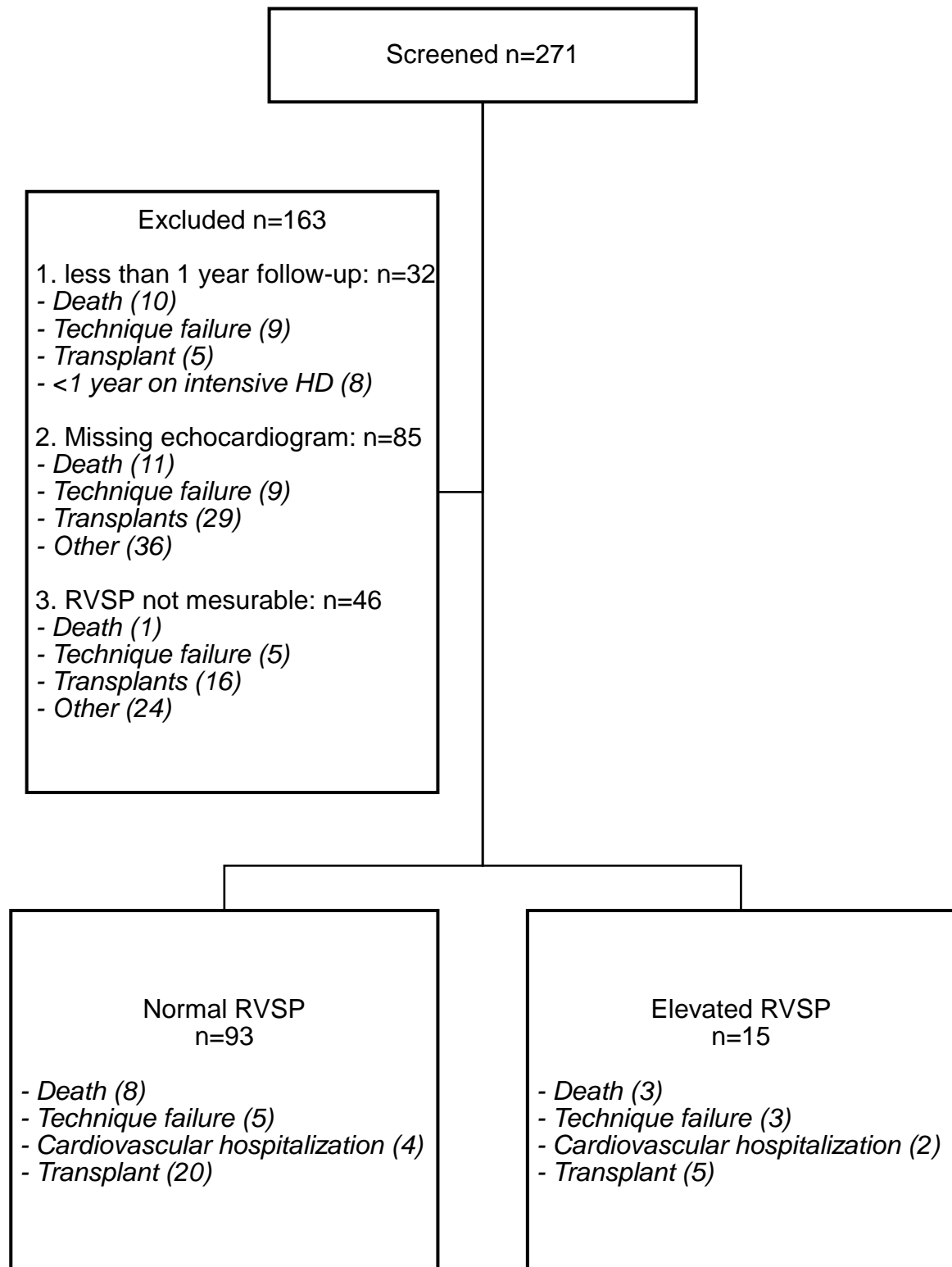

Supplement: Supplementary file 4 — Additional file 4: Figure S1. Flow diagram (≥40 mmHg). [file 12882_2020_2159_MOESM4_ESM.pdf]

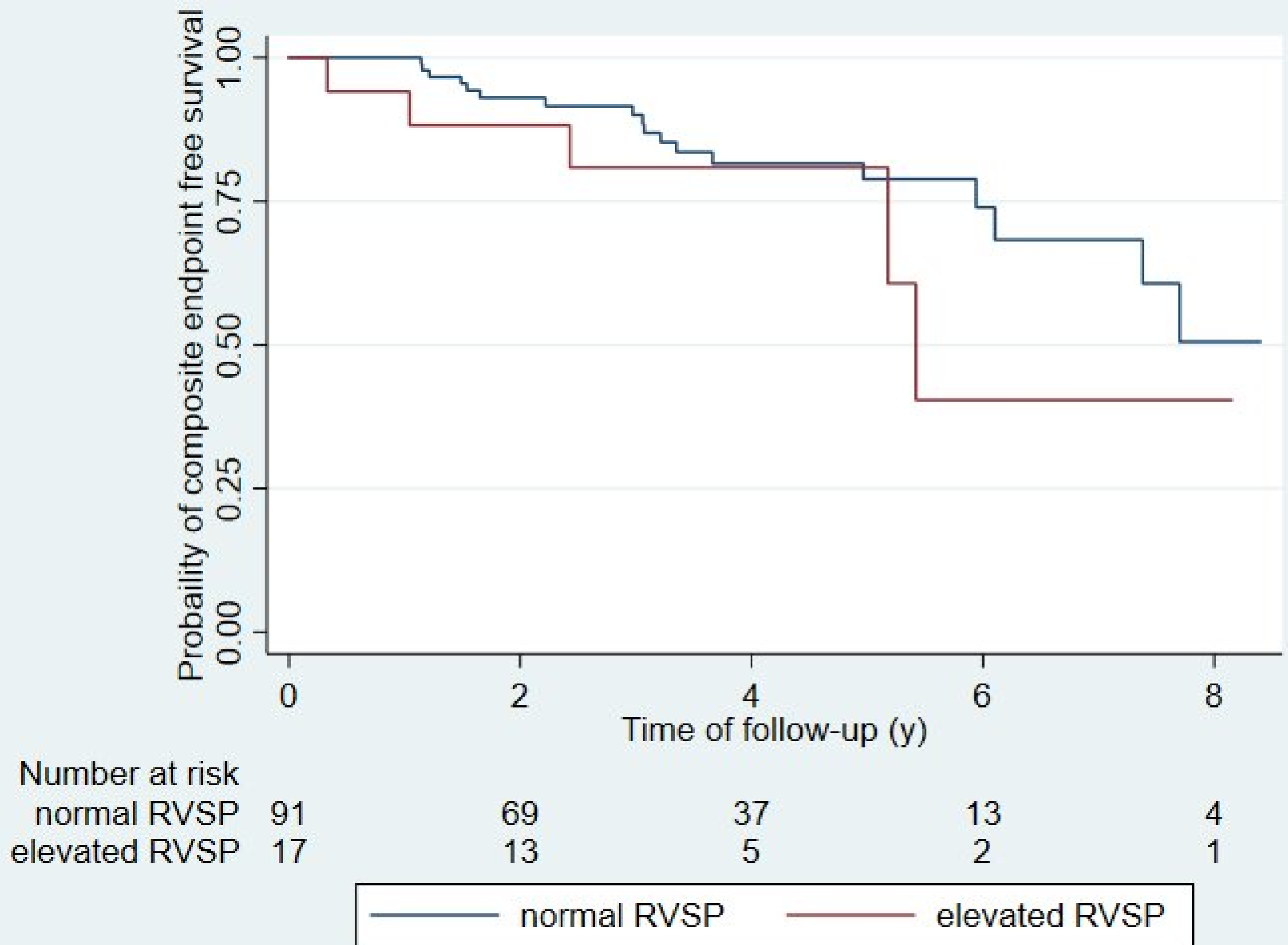

Supplement: Supplementary file 5 — Additional file 5: Figure S2. Composite endpoint free survival in patients with elevated (≥40 mmHg) and normal RVSP at base line, respectively. [file 12882_2020_2159_MOESM5_ESM.pdf]
